# The Stress and Strain of Medicine

**Published:** 1891-07-11

**Authors:** 


### The Hospital

A WEEKLY JOURNAL OF

**Science, Medicine, Nursing, and Philanthropy.**

*For Hospitals, Asylums, Infirmaries, Dispensaries, Medical Practitioners, Students, Nurses, and the Charitable Public.*

VOL. X.—No. 250.] [JULY 11, 1891.

### THE STRESS AND STRAIN OF MEDICINE.

THE tragic death by his own hand of Dr. Charles Edward Sheppard, of Welbeck Street, last week, is a melancholy "sign of the times," to those who belong to the esoteric circle of medical practice in the London of to-day. Dr. Sheppard was but thirty-five years of age, and as a medical man was, therefore, at the very outset of his career. He was a Doctor of Medicine of the University of London and a Gold Medallist, he was also a Fellow of the Royal College of Surgeons of England, and had other professional distinctions. In his capacity as an anæsthetist, Dr. Sheppard was last Tuesday week engaged in administering chloroform to a young lady on whom a delicate operation was about to be performed. He noticed a sudden change in the condition of the patient which alarmed him, and he at once called in his medical colleagues, but death had already taken place. Dr. Sheppard was profoundly affected by this terrible catastrophe, and returned to his consulting rooms in a state of great agitation. Later in the day he was found dead, with a bottle and a glass at his side which had contained prussic acid. At the inquest which was subsequently held, the medical testimony was to the effect that death had resulted from prussic acid poisoning. The coroner, in his summing-up, said that the deceased who was an expert in the administration of chloroform, had no doubt experienced a great shock on the death of the young lady, and that his own death was doubtless due to his own act at a time when his mind had become unhinged.

It ought not to be expected that medical men will be more proof against the temptations to suicide than any other class. But Dr. Sheppard was engaged in the performance of his common and every-day professional duty; and though that duty involved the risk of sudden death to the patient, yet the operator, if he had been in his normal mind, would have felt himself endowed with a courage equal to the occasion. It is the ordinary business of medical men to take upon themselves responsibilities which include questions of life and death. Physicians and surgeons should above all things be men of nerve and courage. They must be prepared to stand firmly by their own judgment and acts, and to face all the possible consequences which may arise even though those consequences may sometimes include among them professional ruin. Not seldom a medical man has to stand in the dock and answer a criminal charge when his sole offence has been the exercise of his best judgment, and a desire to serve the highest interests of his patient.

It is clear that one of the fittest of a doctor's equipments is a nerve of iron. It is equally clear that the physician or surgeon who dares to enter upon the higher walks of the profession ought to be a man who is either blind to remote consequences or who dares to defy them. For it must be remembered that professional ruin is the very worst calamity which can come to a man; it is infinitely worse than death. The soldier takes his life in his hand when he goes into battle; but if his valour should lead him to posts of danger, and if danger should bring death, the death itself often confers a glory and a distinction which prolonged life might entirely fail to achieve. But for a medical man the death of reputation means always the temporary eclipse, and some times even the final blotting out of the sun of hope, and the fatal extinction of the career. If Dr. Sheppard had been himself he would not have laid a hand upon his own life. He would have felt that the terrible accident with which he was so immediately associated was, after all, but an accident, and that he owed it to himself and to his character for courage and manhood to live and work; and so to live and work that he should ultimately prove to the world his capacity as a physician and his trustworthiness as a man.

We do not know what state of bodily health Dr. Sheppard was in before the operation. There is no one to tell us what was the effect of the arduous studies which he had undertaken in the gaining of his professional degrees and distinctions. It is certain, however, that many medical students almost entirely destroy brain and muscle and digestive power in their prolonged efforts to gain high academical honours. Very frequently the result of this is that when they come to the actual duties of their profession, duties which they ought to practise with increasing pleasure, confidence, and success for forty or fifty years, they have no nerve left, no mental strength and steadiness, no high and indomitable courage. They are trembling, fearful, and palsied old young men. Such men, because they have no wise guidance from their teachers during their college life, destroy their health, and with it all possibility of future success and honour. The destruction of their intellectual and physical powers is ruin to themselves, an injury to their families, and an irreparable loss to medicine. Is it possible to make medical teachers and examiners see this? We fear not! Almost all men, being intrinsically small men, magnify their office. If we could get a class of medical teachers and examiners who had mental capacity enough to be greater than their office we might have hope.

There are certain facts to be reckoned with at the present time which the members of the medical profession do not seem to have become intelligently conscious of. The world makes larger and sterner demands upon medicine than it ever made before. Not only so, but it makes sterner demands upon medicine than it does upon any other profession. The eye that is turned upon theology, and even upon law, is mild and benevolent compared with the piercing and critical gaze which is directed upon every act of medicine, both in the narrow field of the home and in the great outside world. There are two new sets of critics with which doctors have to deal—the newspaper man and the trained nurse. The trained nurse knows little of physiology or pathology, but she understands clearly and well the upward or downward progress of her patient. Although she cannot picture to herself what is transpiring in the organs of the sufferer's body as he lies upon his bed, she can follow with the most subtle intuition the processes which are being carried on in the physician's mind. She knows with the utmost certainty whether he reasons weakly or strongly, wisely or inconsequently; and whether the general drift of his treatment is to aid the patient on his road towards health, or merely to leave him to struggle as best he can with such help as Nature gives.

The newspaper man is to the doctor in the great world what the nurse is in the home: he is a half-instructed critic. He knows when a patient is cured; he judges solely by gross results. Of physiology and pathology, of the consequences of physical wear and tear, brain worry, and the keen life struggle, to the human organism, he knows nothing at all. But he sits in judgment all the same. The sensitive and nerve-weakened doctor, who has ruined his own physique in the effort to discover new ways and means of preserving the life and physique of other people, feels that from an unhappy misadventure there is no appeal either to a generous public or to an intelligent press. It is a cruel position: a position which such men as Dr. Sheppard cannot endure. They kill themselves.

The medical profession has itself solely to blame for this intolerable state of things. It has persistently laboured to keep the public in total ignorance both of the science and the art of medicine. Time has brought its revenges. The public, though it knows nothing, insists upon judging, and its judgment often means death to the merely unlucky, but life and wealth to the unscrupulous and the bold. If Dr. Sheppard had known that the public and the press were in a position to form an intelligent judgment of the merits of his case, he would in all probability have been alive to-day. Those medical men who insist upon carrying on their work in the obscure recesses of the temple of mystery are the most dangerous of all the enemies which the medical profession has to fear.

